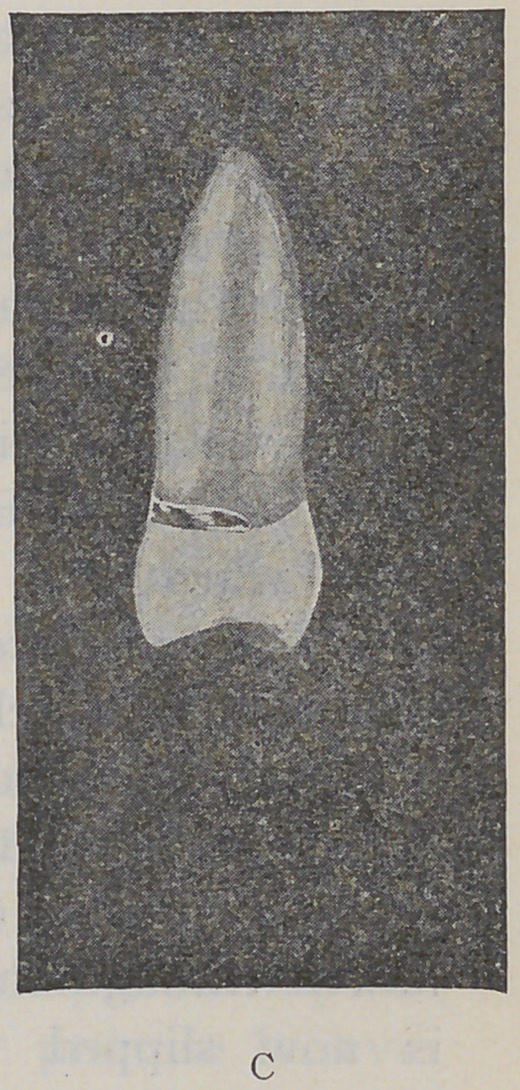# A New Porcelain Crown

**Published:** 1903-10-15

**Authors:** J. P. Carmichael


### A NEW PORCELAIN CROWN.

BY J. P. CARMICHAEL, D.D.S.

I present herewith a plan of making a porcelain crown which I believe excels in technique and is hygienic and esthetic, and can be applied to nearly all cases and conditions. By the use of this methcd the objectionable band on the labial or buccal surface is done away with, and the porcelain facing is molded upon the end of the root, with the nice adaptation of a porcelain inlay.

In constructing this crown I make use of the rolled crown post, which not only gives the adaptation, but makes an attachment for the crown that will not move from its position while getting the form for the inlay or labial portion; it also throws the required strength for the support of the crown into the post. The crown post is a hollow cone made by rolling a spiral of thin platinum of desired size and length.

The metal of this post is easily and perfectly adapted to the form of the root-canal, and the projecting end may be shaped to form a perfect seat for the crown upon the root. The least possible cement is required in setting; the half band serves to protect that portion of the root from decay and fracture from the force of mastication.

I first make a narrow band of rather thin platinum about 32 gauge, and solder on a top of platinum, about 36 gauge; this cap is then placed on the root, and with a sharp instrument punch through it into the root-canal; this opening is then enlarged with a blunt instrument until the hole is the full size of the root-canal, with the edge of the metal curled down into the root and against the walls of the canal, while the cap is resting firmly on the end of the root.

The post should be of sufficient length for the canal, leaving enough projecting to cover the cap. The post is first placed in the root and with blunt instruments it is easily enlarged to the shape and size of the root-canal, and the projecting end rubbed down upon and over the cap; a second post, a little shorter than the first, is then used in the same manner, and if necessary a third one, each telescoping the other. By beginning with the long post, and finishing with the short ones, the solder will flow entirely through the coil. This may be facilitated by making a pin-hole through to the end of the post with a sharp-pointed instrument which will permit the solder to flow to the end.

I then remove the cap with the post which remains firmly in its position in the cap, fill the post with small pieces of platinum solder and flow. The solder will flow through the coiled metal, and makes a very rigid post; the cap and the post are thus united in one piece. The post is usually left hollow, but can be entirely filled with platinum solder. But the hollow post may provide a convenient place to set a platinum support for the porcelain.

The porcelain facing is now fitted in its proper position without grinding it closely to the root and not soldered to the attachments. The cap and band are now cut away as far back as the post from the labial surface. The cap will then present the form shown in the upper drawing seen in cut "A."

To get the adaptation of the porcelain to the end of the root, take a piece of thin inlay platinum of sufficient size to cover the end of the root and to lap a little over the buccal edge; punch a hole through this, the size of the crown post, and slide it down into the cap over the post. This is now placed upon the root and forced down to position,

A B C

burnishing the thin platinum disc to the end of the root, fitting the form over the edge of the root and under the free margin of the gum. The facing is then adjusted to position and fastened as seen in cut "B." I then remove the crown with the foil in place, which is held firmly in position by the post. If necessary a little wax can be used to hold the foil in place as it will burn out in the baking.

Proceed in the usual manner of filling in with porcelain body and baking. The finished crown is shown in cut "C." When the crown is completed, peel off the platinum foil, and you have a most accurate adaptation to the root at the gum margin, which provides a sufficient amount of porcelain at this juncture to prevent any liability of fracture or checking.

As time is a great factor in dental operations, I will describe in this connection a porcelain crown for the six front teeth, made by using the rolled crown post, and adjusting the porcelain facing upon the natural root, similar to the bicuspid illustrated in figure "B." Place a properly selected rolled post in the root-canal and after enlarging it to the root-canal, spin the projecting end down upon the root and over the palatal edge to the gum margin, thus forming a half cap. Insert in the first post a second one and burnish it down over the first in the same manner, and then insert a third post, allowing it to stand out far enough to receive the pins of the porcelain facing. Place the porcelain facing in position with the pins astride the standing post. Put a small platinum bar into the rolled post, which will prevent crushing the post and provide sufficient metal to give the required strength. The pins may now be clinched around the post and the whole drawn off the root united by the close adaptation.

An investment is used to protect the porcelain, leaving the post entirely exposed to the heat, and platinum solder flowed through the entire mass. A disc of inlay platinum is now slipped over the post and the crown placed upon the root and forced to position; the platinum disc is now burnished to the labial face of the root and the crown carefully removed from the root. It is now ready for baking, after which remove the disc of platinum and you have a crown perfectly fitted to the root. A crown can be constructed in this way quickly and so well adapted that it will have many desirable qualities and will meet the requirements of cases which other methods would not.

## Figures and Tables

**Figure f1:**
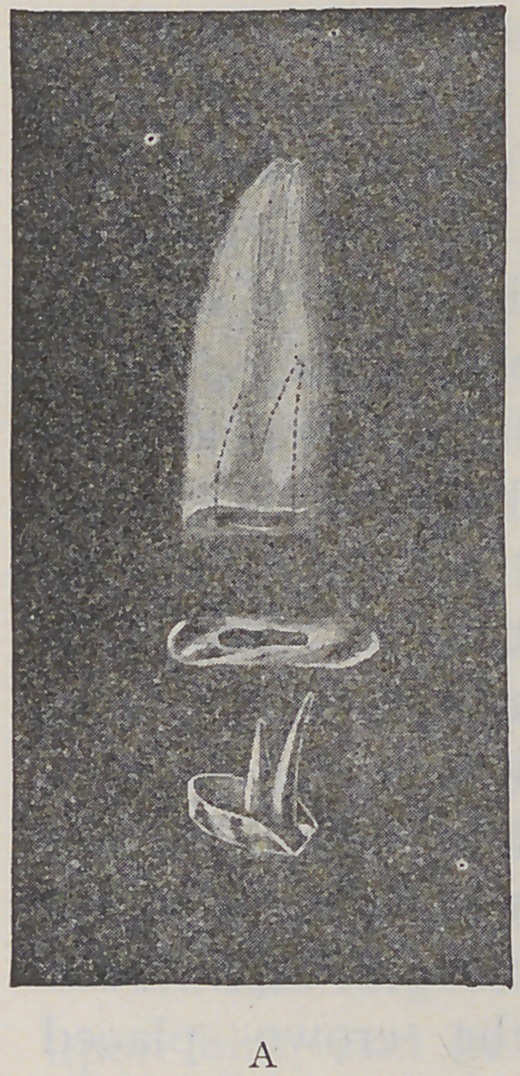


**Figure f2:**
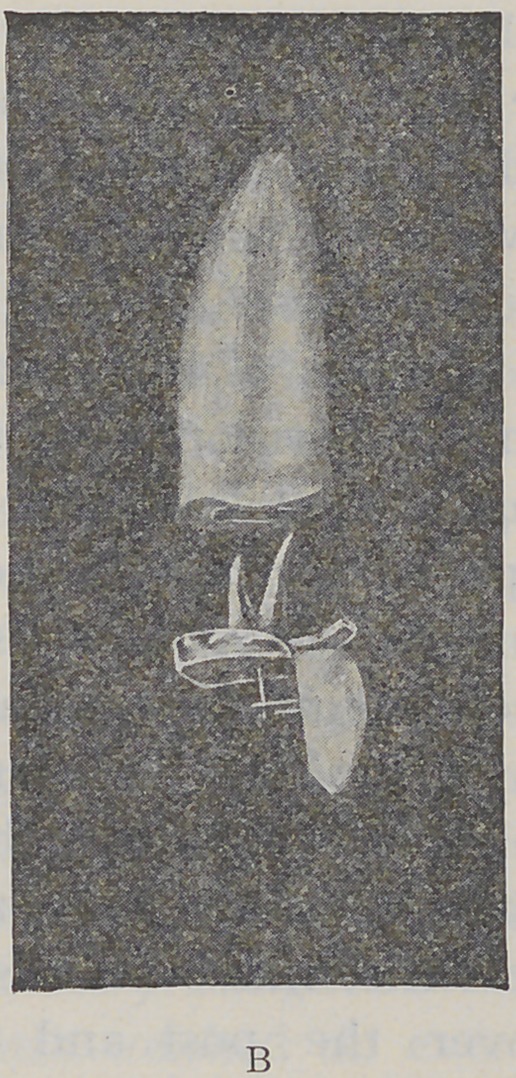


**Figure f3:**